# Establishment and validation of a prognostic nomogram for extrahepatic cholangiocarcinoma

**DOI:** 10.3389/fonc.2022.1007538

**Published:** 2022-11-24

**Authors:** Fangrui Zhao, Dashuai Yang, Jiahui He, Xianli Ju, Youming Ding, Xiangpan Li

**Affiliations:** ^1^ Department of Oncology, Renmin Hospital of Wuhan University, Wuhan, Hubei, China; ^2^ Department of Hepatobiliary Surgery, Renmin Hospital of Wuhan University, Wuhan, Hubei, China; ^3^ Department of Pathology, Renmin Hospital of Wuhan University, Wuhan, Hubei, China

**Keywords:** extrahepatic cholangiocarcinoma, AJCC staging system, nomogram, prognostic model, risk stratification, cancer-specific survival

## Abstract

**Simple summary:**

Accurately estimate the prognosis of patients with ECCA is important. However, the TNM system has some limitations, such as low accuracy, exclusion of other factors (e.g., age and sex), and poor performance in predicting individual survival risk. In contrast, a nomogram-based clinical model related to a comprehensive analysis of all risk factors is intuitive and straightforward, facilitating the probabilistic analysis of tumor-related risk factors. Simultaneously, a nomogram can also effectively drive personalized medicine and facilitate clinicians for prognosis prediction. Therefore, we construct a novel practical nomogram and risk stratification system to predict CSS in patients with ECCA.

**Background:**

Accurately estimate the prognosis of patients with extrahepatic cholangiocarcinoma (ECCA) was important, but the existing staging system has limitations. The present study aimed to construct a novel practical nomogram and risk stratification system to predict cancer-specific survival (CSS) in ECCA patients.

**Methods:**

3415 patients diagnosed with ECCA between 2010 and 2015 were selected from the SEER database and randomized into a training cohort and a validation cohort at 7:3. The nomogram was identified and calibrated using the C-index, receiver operating characteristic curve (ROC), and calibration plots. Decision curve analysis (DCA), net reclassification index (NRI), integrated discrimination improvement (IDI) and the risk stratification were used to compare the nomogram with the AJCC staging system.

**Results:**

Nine variables were selected to establish the nomogram. The C-index (training cohort:0.785; validation cohort:0.776) and time-dependent AUC (>0.7) showed satisfactory discrimination. The calibration plots also revealed that the nomogram was consistent with the actual observations. The NRI (training cohort: 1-, 2-, and 3-year CSS:0.27, 0.27,0.52; validation cohort:1-,2-,3-year CSS:0.48,0.13,0.34), IDI (training cohort: 1-, 2-, 3-year CSS:0.22,0.18,0.16; validation cohort: 1-,2-,3-year CSS:0.18,0.16,0.17), and DCA indicated that the established nomogram significantly outperformed the AJCC staging system (*P*<0.05) and had better recognition compared to the AJCC staging system.

**Conclusions:**

We developed a practical prognostic nomogram to help clinicians assess the prognosis of patients with ECCA.

## Introduction

Cholangiocarcinoma (CCA) is a highly invasive malignant tumor originating from bile duct epithelial cells, and it is the second most common primary liver malignancy after hepatocellular carcinoma (HCC), accounting for approximately 3-5% of gastrointestinal malignancies (1). Depending on the anatomical location of origin, CCA is usually divided into intrahepatic CCA (ICCA), perihilar CCA (pCCA), and distal CCA (dCCA). Perihilar CCA and distal CCA are commonly referred to as extrahepatic cholangiocarcinoma (ECCA), accounting for 70–90% of all CCA cases, while ICCA accounts for only 10-20% (2). Several studies have shown noticeable differences in the prognosis of ICCA and ECCA, suggesting that ECCA should be studied independently rather than conducting a general analysis without identifying the anatomical sites. Based on the anatomical location of CCA, careful assessment of prognosis is essential (3–6).

The American Joint Committee on Cancer (AJCC) tumor-node-metastasis (TNM) system is the most commonly used method to evaluate the prognosis of patients with ECCA (7, 8). However, the TNM system has some limitations, such as low accuracy, exclusion of other factors (e.g., age and sex), and poor performance in predicting individual survival risk (9). As a result, a new and personalized prediction model is needed to evaluate the prognosis of ECCA patients.

A nomogram-based clinical model related to a comprehensive analysis of all risk factors has been widely used in tumor patients to predict survival (10–12). More importantly, a nomogram is intuitive and straightforward, facilitating the probabilistic analysis of tumor-related risk factors. Simultaneously, a nomogram can also effectively drive personalized medicine and facilitate clinicians for prognosis prediction (13–15). In the present study, we aimed to develop a nomogram and risk stratification system for patients with ECCA by utilizing a large dataset from SEER (Surveillance, Epidemiology, and End Results).

## Materials and methods

### Data sources

Clinically relevant data of patients diagnosed with ECCA between 2010 and 2015 were extracted from the SEER 18 registry database (1975–2018) using SEER*Stat 8.3.9.2. International Classification of Diseases (ICD) for Oncology C24.0 (ICD-O C24.0) and ICD code O-3 morphology (8032, 8033, 8070, 8071,8140, 8141, 8160, 8161, 8162, 8260, 8480, 8481, 8490, and 8560) were used to make the distinction. The following 13 variables were included from the SEER database: age (at diagnosis), ethnicity, sex, marital status (at diagnosis), insurance, tumor number, infiltration, tumor size, lymph node status, tumor stage (AJCC stage), surgery, radiotherapy, and chemotherapy. In addition, the seventh edition of the AJCC staging system was used for the analysis. The SEER database was publicly accessible, and private data for all patients were removed from the database, indicating that Institutional Review Board approval and informed consent were not needed.

### Selection criteria

**Figure 1:**
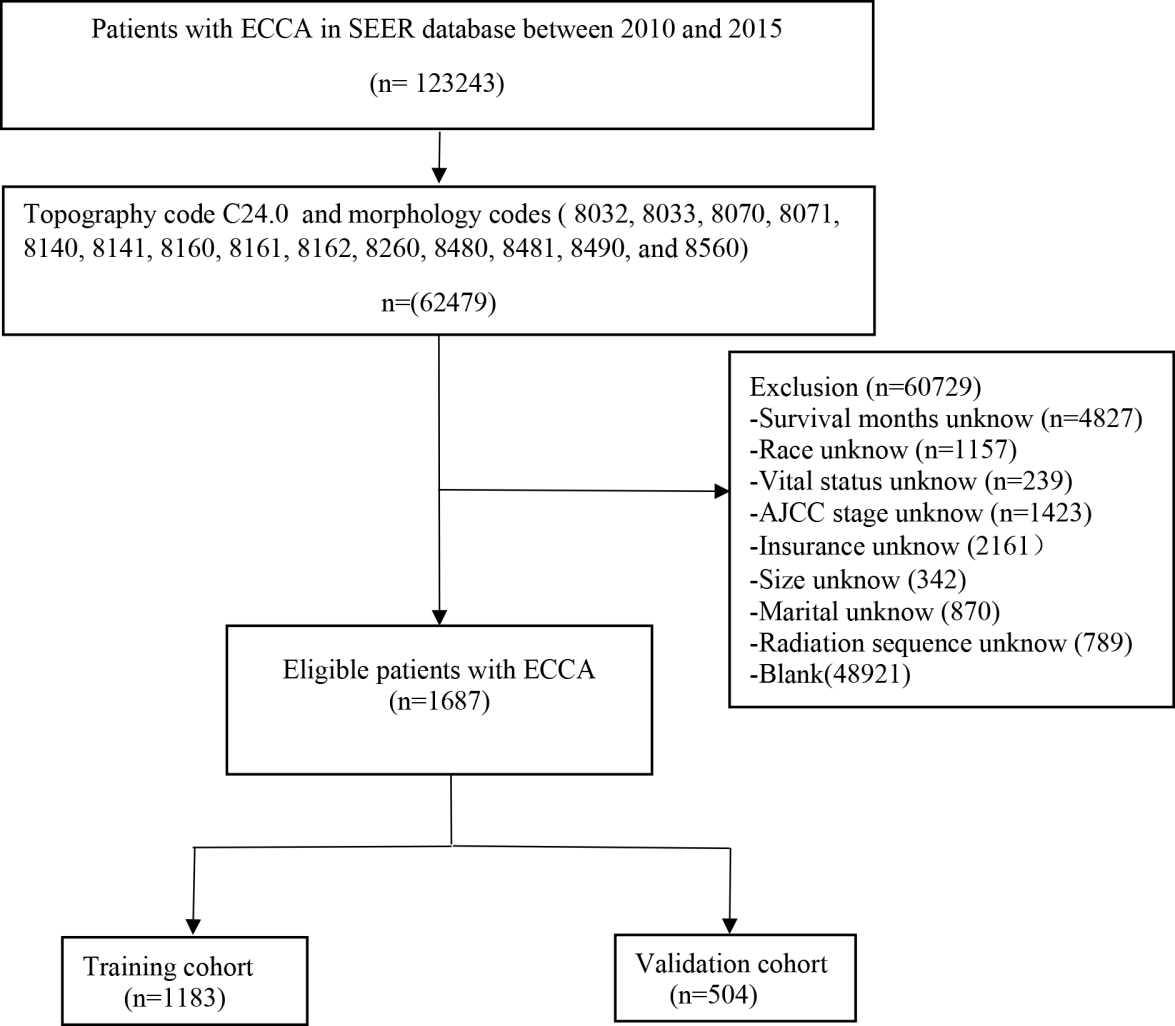
Flow diagram of the ECCA patients in the training and validation cohorts. ECCA: extrahepatic cholangiocarcinoma.

The inclusion criteria were as follows: (a) patients with ECCA (topography code C24.0 and morphology codes 8032, 8033, 8070, 8071, 8140, 8141, 8160, 8161, 8162, 8260, 8480, 8481, 8490, and 8560); (b) confirmed AJCC staging; (c) complete treatment information; and (d) complete follow-up information. The exclusion criteria were as follows: (a) unknown primary location of the tumor; (b) incomplete follow-up information; (c) incomplete treatment information; (d) unknown AJCC staging; and (e) unknown tumor size. The flow chart in **Figure 1** shows the process of screening.

### Construction of the nomogram

Study cohorts listed the clinical characteristics of ECCA. All patients were randomized into a training cohort (n=1183, 70%) and a validation cohort (n=504, 30%). The training cohort was used to filter the variables and build the model, while the validation cohort was used to verify the results. Univariate and multivariate Cox regression analyses were also performed to screen unique variables that significantly affected cancer-specific survival (CSS) in ECCA and were applied to construct the nomogram. Variables with *P*<0.05 in both univariate and multivariate Cox regression were considered independent risk factors.

### Validation of the nomogram

The consistency index (C-index), time-dependent receiver operating characteristic curve (ROC), calibration curve, and decision curve analysis (DCA) were used to verify the nomogram. The C-index was used to reflect the performance prediction accuracy of the nomogram, while the ROC represented the sensitivity and specificity of the nomogram. Generally, 0.50 to 0.70 indicated low accuracy, 0.71 to 0.90 indicated moderate accuracy, and greater than 0.90 indicated high accuracy. We plotted 1-, 2-, and 3-year calibration curves to compare the predicted CSS with that observed in our model, and we used the 45-degree line as the actual outcome of the primary model.

### Comparison between the risk stratification associated with the nomogram and AJCC staging system

The net reclassification index (NRI), C-index, integrated discrimination improvement (IDI), and DCA were used to assess the nomogram model’s net benefit and risk stratification compared to the AJCC staging system. The clinical utility of the nomogram was evaluated by DCA. All eligible patients were divided into three groups, namely, the low-risk group, middle-risk group, and high-risk group, with the best cutoff value for the total score selected by X-Tile. The Kaplan–Meier curve and log-rank test were performed to compare CSS in different groups of patients.

### Statistical analysis

All statistical analyses were performed using R Software Version 4.1.2 (http://www.r-project.org/). The “regplot”, “mstate”, “survival”, “cmprsk”, “Hmisc”, “timeROC”, “foreign”, “nricens”, “rmda”, and “DCA” packages in R were used to develop and verify the nomogram. Statistical distribution differences between the training and validation cohorts were analyzed using the chi-square test. The variance inflation factor was applied to assess the detection of multicollinearity testbetween variables. All *P* values were two-tailed, and *P*< 0.05 was considered statistically significant.

## Results

### Characteristics of patients

A total of 3415 patients were identified to have ECCA, and they were randomized into a training cohort and a validation cohort at a ratio of 7:3. The median follow-up times for the entire population, training cohort, and validation cohort were 12 months, while the interquartile ranges (IQRs) were 4-23, 4-24, and 4-22 months, respectively. The demographic and clinical characteristics of patients with ECCA are summarized in **Table 1**. A total of 946 male patients and 741 female patients accounted for 56.08% and 43.92%, respectively. There were 1284 Caucasians and 126 African Americans, which accounted for 76.11% and 7.47%, respectively. Of all the patients, 812 (48.13%) did not have surgery, 455 (26.97%) underwent liver resection, and 420 (24.90%) underwent liver transplantation. Only 834 patients (49.44%) received chemotherapy. Additionally, 301 (17.84%) patients received radiotherapy. The percentages of married and insured patients were 60.40% and 83.11%, respectively. The training and validation cohorts were comparable in terms of demographic and clinical characteristics (*P*>0.05).

**Table 1:** Demographics and clinical characteristics of ECCA at diagnosis.

Variable	Whole population		Training cohort		Validation cohort		*P* Value
	n	%	n	%	n	%
	1687	100	1183	100	504	100	
Age							
<65	685	40.60	493	3.80	210	41.67	0.83
≥65	1002	59.40	675	59.85	249	49.40	
Race							
Black	126	7.47	92	7.78	34	6.75	0.74
White	1284	76.11	874	73.88	374	74.21	
Other	313	18.55	217	18.34	96	19.05	
Sex							
F	741	43.92	545	46.07	237	38.89	0.71
M	946	56.08	638	53.93	267	61.11	
AJCC Stages ^a^							
I	518	30.71	353	29.84	165	32.74	0.54
II	412	24.42	318	26.88	124	24.60	
III	216	12.80	180	15.22	81	16.07	
IV	466	27.62	332	28.06	134	26.59	
Grade ^b^							
Well	583	34.56	413	34.91	170	33.73	0.88
Bad	402	23.83	279	23.58	123	24.40	
Unknown	702	41.61	491	41.50	211	41.87	
Size							
0-2 cm	645	38.23	458	38.72	187	37.10	0.81
2-5 cm	801	47.48	558	47.17	243	48.21	
>5 cm	241	14.29	167	14.12	74	14.68	
Number							
1	1604	95.08	1122	94.84	482	95.63	0.49
>1	83	4.92	61	5.16	22	4.37	
Regional nodes status							
Negative	444	26.32	34	2.87	130	25.79	0.64
Not examined	795	47.13	549	46.41	246	48.81	
Positive	448	26.56	320	27.05	128	25.40	
Treatment							
No operation	812	48.13	567	47.93	245	48.61	0.34
Liver resection	455	26.97	330	27.90	125	24.80	
Transplant	420	24.90	286	24.18	134	26.59	
Radiation sequence							
No radiation	1416	83.94	991	83.77	425	84.33	0.86
After surgery	292	17.31	185	15.64	77	15.28	
Prior to surgery	9	0.53	7	0.59	2	0.40	
Chemotherapy							
Yes	834	49.44	579	48.94	255	50.60	0.53
No	853	50.56	604	51.06	249	49.40	
Marital							
Married	1019	60.40	716	60.52	303	60.12	0.98
Divorced	427	25.31	299	25.27	128	25.40	
Single	241	14.29	168	14.20	73	14.48	
Insurance							
Insured	1402	83.11	984	83.18	418	82.94	0.13
Uninsured	50	2.96	29	2.45	21	4.17	
Any Medicaid	235	13.93	170	14.37	65	12.90	

^a^The seventh edition American Joint Committee on Cancer (AJCC) TNM staging system. ^b^-Well: Grade I and Grade II; Bad: Grade III and Grade IV. ECCA: extrahepatic cholangiocarcinoma.

### Univariate and multivariate Cox regression analyses

The variance inflation factors (1.126-3.521) were all less than 5 indicating that there was no collinearity between the variables (**Supplemental Table 2**). Univariate and multivariate Cox regression analyses suggested that age, AJCC staging, pathological grade, lymph nodes, treatment, chemotherapy, tumor size, tumor number, and marital status were independent prognostic factors (*P*<0.05) and were included in constructing the nomogram (**Table 2**).

**Table 2:** The results of univariate and multivariate Cox regression analyses on variables for the prediction of CSS.

Variable	Univariate analysis		*P* Value	Multivariate analysis		*P* Value
	HR	95% CI		HR	95% CI
Age						
<65	Reference			Reference		
≥65	1.26	1.09-1.44	<0.001	1.17	1.01-1.36	<0.001
Race						
Black	Reference			Reference		
White	0.74	0.59-0.94	<0.001	0.79	0.62-1.12	0.05
Other	0.72	0.55-0.94	<0.001	0.76	0.58-1.01	0.06
Sex						
Female	Reference			Reference		
Male	0.82	0.72-0.94	<0.001	0.98	0.85-1.12	0.79
Grade ^a^						
Well	Reference			Reference		
Bad	1.3	1.08-1.56	<0.001	1.26	1.04-1.52	0.01
Unknow	2.81	2.40-3.28	<0.001	1.06	0.86-1.31	0.62
Regional nodes status						
Negative	Reference			Reference		
Positive	1.51	1.24-1.83	<0.001	1.6	1.29-1.99	<0.001
Unknow	3.69	3.11-4.39	<0.001	1.72	1.34-2.21	<0.001
AJCC Stages ^b^						
I	Reference			Reference		
II	0.98	0.81-1.17	0.351	1.39	1.12-1.72	<0.001
III	1.34	1.09-1.66	<0.001	1.28	1.02-1.61	<0.001
IV	2.7	2.27-3.21	<0.001	1.97	1.62-2.40	<0.001
Size						
0-2 cm	Reference			Reference		
3-5 cm	1.35	1.17-1.55	<0.001	1.23	1.06-1.42	<0.001
>5 cm	1.96	1.61-2.39	<0.001	1.39	1.13-1.71	<0.001
Number						
1	Reference			Reference		
>1	0.5	0.36-0.69	<0.001	0.52	0.37-0.73	<0.001
Treatment						
No operation	Reference			Reference		
Hepatectomy	0.28	0.23-0.33	<0.001	0.39	0.30-0.52	<0.001
Transplant	0.25	0.21-0.30	<0.001	0.34	0.25-0.46	<0.001
Radiation sequence						
No	Reference			Reference		
After surgery	0.44	0.36-0.54	<0.001	0.98	0.77-1.23	0.86
Prior to surgery	0.82	0.39-1.73	0.18	1.76	0.81-3.79	0.14
Chemotherapy						
Yes	Reference			Reference		
No	1.56	1.37-1.786	<0.001	2.09	1.79-2.44	<0.001
Marital						
Married	Reference			Reference		
Divorced	1.59	1.37-1.85	<0.001	1.24	1.05-1.47	<0.001
Single	1.61	1.33-1.94	<0.001	1.24	1.02-1.51	0.02
Insurance						
Insured	Reference			Reference		
Uninsured	0.24	0.21-1.15	0.25	1.45	0.93-2.24	0.09
Any Medicaid	0.14	0.09-1.58	0.11	0.97	0.80-1.18	0.81

^a^Well: Grade I and Grade II; Bad: Grade III and Grade IV; ^b^-The seventh edition American Joint Committee on Cancer (AJCC) TNM staging system. CSS: cancer-specific survival.

### Construction and validation of the nomogram

**Figure 2:**
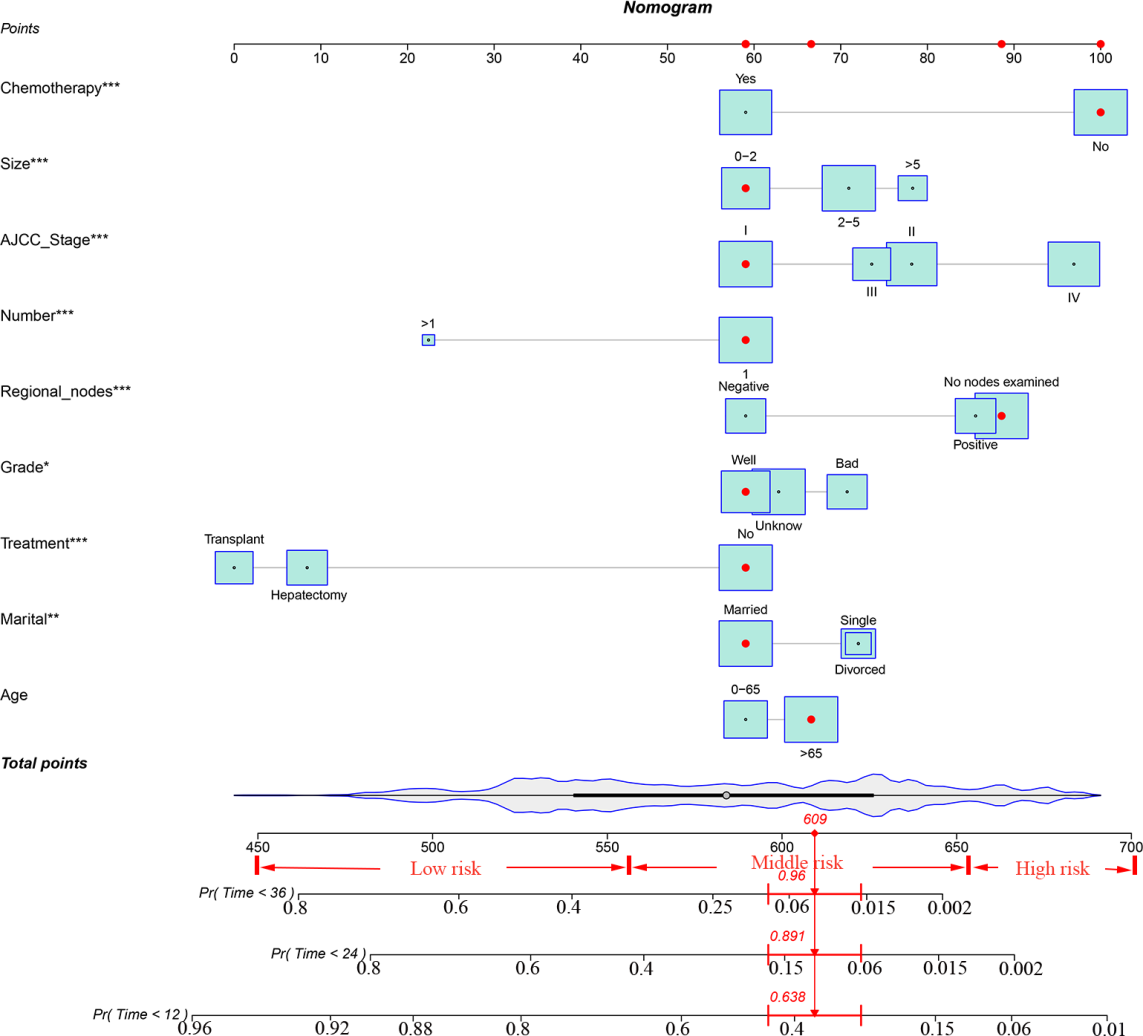
A nomogram for ECCA patients and new risk stratification.

**Figure 3:**
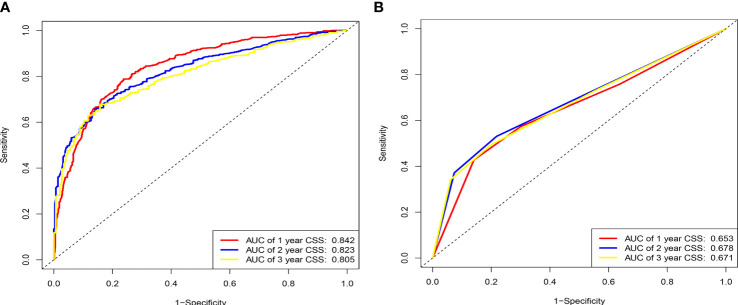
ROC curves for the AJCC staging and nomogram for 1-, 2-, and 3-year prediction. **(A)** Training cohorts based on the nomogram. **(B)** Validation cohorts based on AJCC staging.

**Figure 4:**
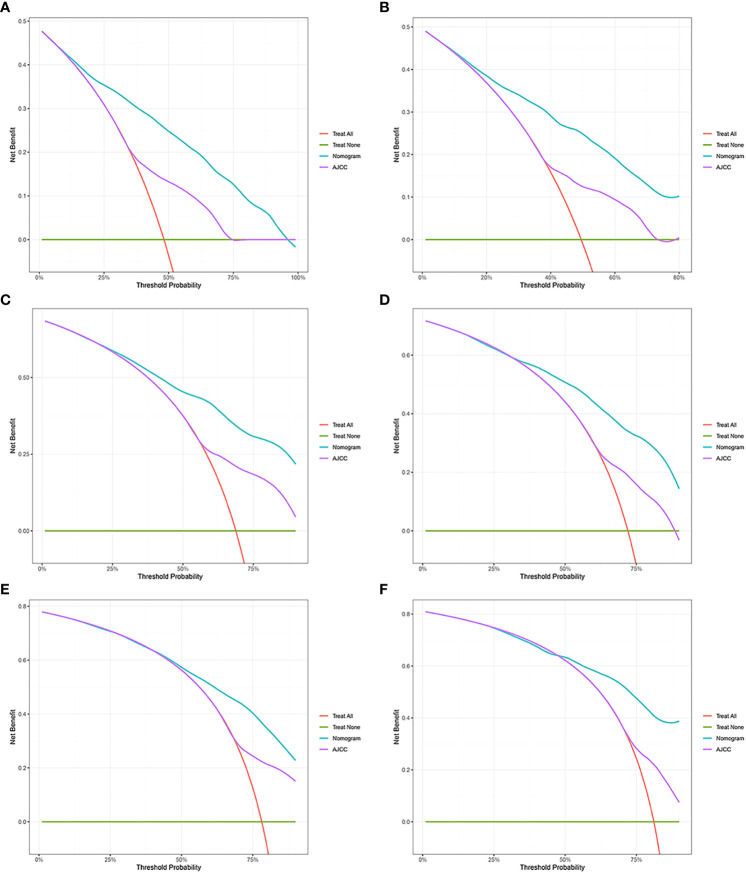
Decision curve analysis. **(A, C, E)** DCA curves of 1-year, 2-year, and 3-year CSS in the training cohort. **(B, D, F)** DCA curves of 1-year, 2-year, and 3-year CSS in the validation cohort. DCA, decision curve analysis; CSS, cancer-specific survival.

**Figure 5:**
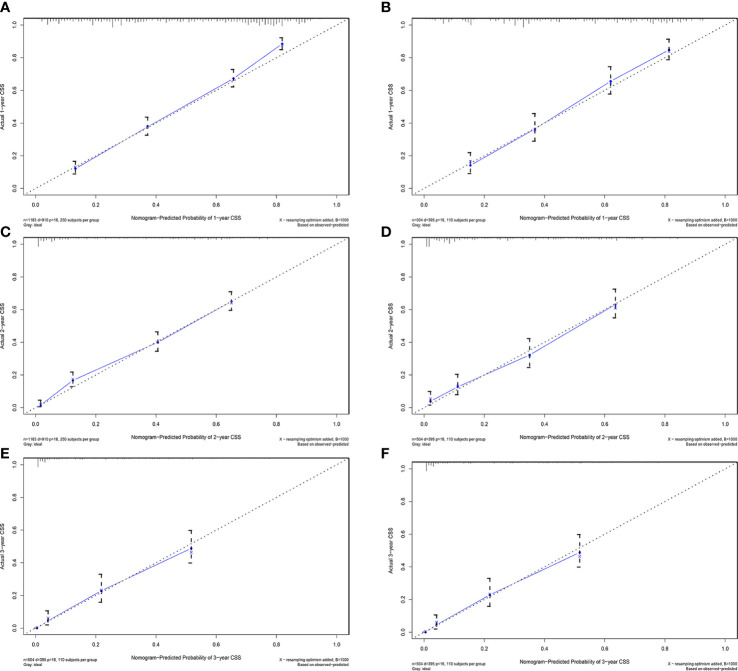
Calibration plots of 1-year, 2-year, and 3-year CSS for ECCA patients. **(A, C, E)** Calibration plots of 1-year, 2-year, and 3-year CSS in the training cohort. **(B, D, F)** Calibration plots of 1-year, 2-year, and 3-year CSS in the training cohort. CSS, cancer-specific survival.

Based on the univariate and multivariate Cox regression analyses, independent prognostic factors were selected to construct the nomogram to predict CSS for patients with ECCA (**Figure 2**). To predict the probability of CSS in patients with ECCA, risk scores for each variable were derived based on patients’ information. Second, all risk scores were added to find the corresponding scores in line with the total scores. Finally, the probability of 1-, 2-, and 3-year CSS for patients with ECCA was determined by drawing a straight line on the last 3 rows.The C-index for the training and validation cohorts was 0.785 (95% CI: 0.741-0.792) and 0.776 (95% CI: 0.716-0.788), respectively. The ROC, and DCA and calibration curves are shown in **Figures 3**–**5**, respectively. The ROC curve showed that the 1-, 2-, and 3-year

AUC values in the training cohort were 0.821, 0.817, and 0.846, respectively. The AUC values at 1 year, 2 years, and 3 years in the validation cohort were 0.829, 0.818, and 0.828, respectively, indicating a good predictive performance of the model. Furthermore, the DCA curves show good clinical application potential and better positive net benefit in the training and validation cohorts. The calibration curves agreed with the predicted CSS rates at 1, 2, and 3 years.

### Clinical value of the nomogram compared to the tumor stage based on AJCC staging

**Figure 6:**
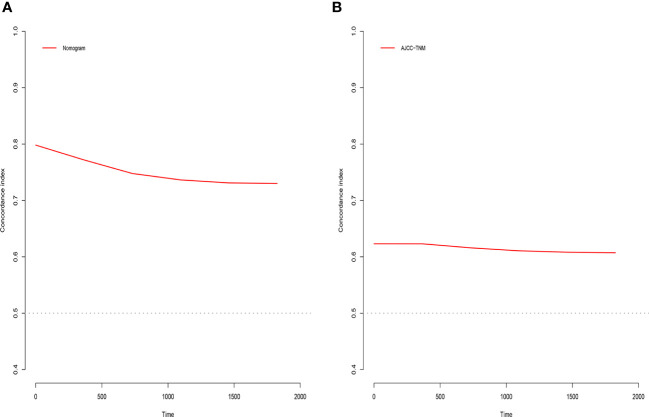
C-index analysis. **(A)** Nomogram-related C-index. **(B)** AJCC staging criteria-related C-index.

The C-index, NRI, ROC, and IDI were used to compare the accuracy between the nomogram and AJCC staging system. In the training cohort, the C-index of the nomogram was higher than that of the AJCC staging system (**Figure 6**). The 1-, 2-, and 3-year NRIs were 0.27 (95% CI=0.14-0.41), 0.27 (95% CI=0.11-0.45), and 0.52 (95% CI=0.41-0.59), respectively (**Table 3**). The 1-, 2-, and 3-year time-dependent ROC curves for the nomogram were 0.842, 0.823, and 0.805, respectively, while those for the AJCC staging system were 0.653, 0.678, and 0.671, respectively, indicating that the model had excellent predictive performance. IDI (training cohort: 1-, 2-, 3-year CSS: 0.22, 0.18, 0.16; validation cohort: 1-, 2-, 3-year CSS: 0.18, 0.16, 0.17) indicated that the established nomogram significantly outperformed AJCC staging system (*P*<0.05) (**Table 3**). The net benefit of the nomogram was compared to that of the AJCC staging system. The DCA curves showed that the nomogram better predicted 1-, 2-, and 3-year CSS in the training and validation cohorts because it added more net benefit than the AJCC staging system. The differences between the AJCC staging system and nomogram was shown in **Supplemental Table 1**.

**Table 3:** NRI and IDI of the nomogram and AJCC staging criteria alone in CSS prediction for ECCA.

Index		Training cohort		*P* value	Validation cohort		*P* value
		Estimate	95% CI		Estimate	95% CI
**NRI**	For 1-year CSS	0.27	0.14-0.41		0.48	0.30-0.61	
	For 2-year CSS	0.27	0.11-0.45		0.13	0.07-0.29	
	For 3-year CSS	0.52	0.41-0.59		0.34	0.26-0.47	
**IDI**	For 1-year CSS	0.22	0.17-0.26	<0.001	0.18	0.13-0.23	<0.001
For 2-year CSS	0.18	0.15-0.22	<0.001	0.16	0.10-0.24	<0.001
For 3-year CSS	0.16	0.12-0.20	<0.001	0.17	0.10-0.25	<0.001

ECCA, extrahepatic cholangiocarcinoma; CSS, cancer-specific survival.

### Establishment of a stratified risk system based on the nomogram

**Figure 7:**
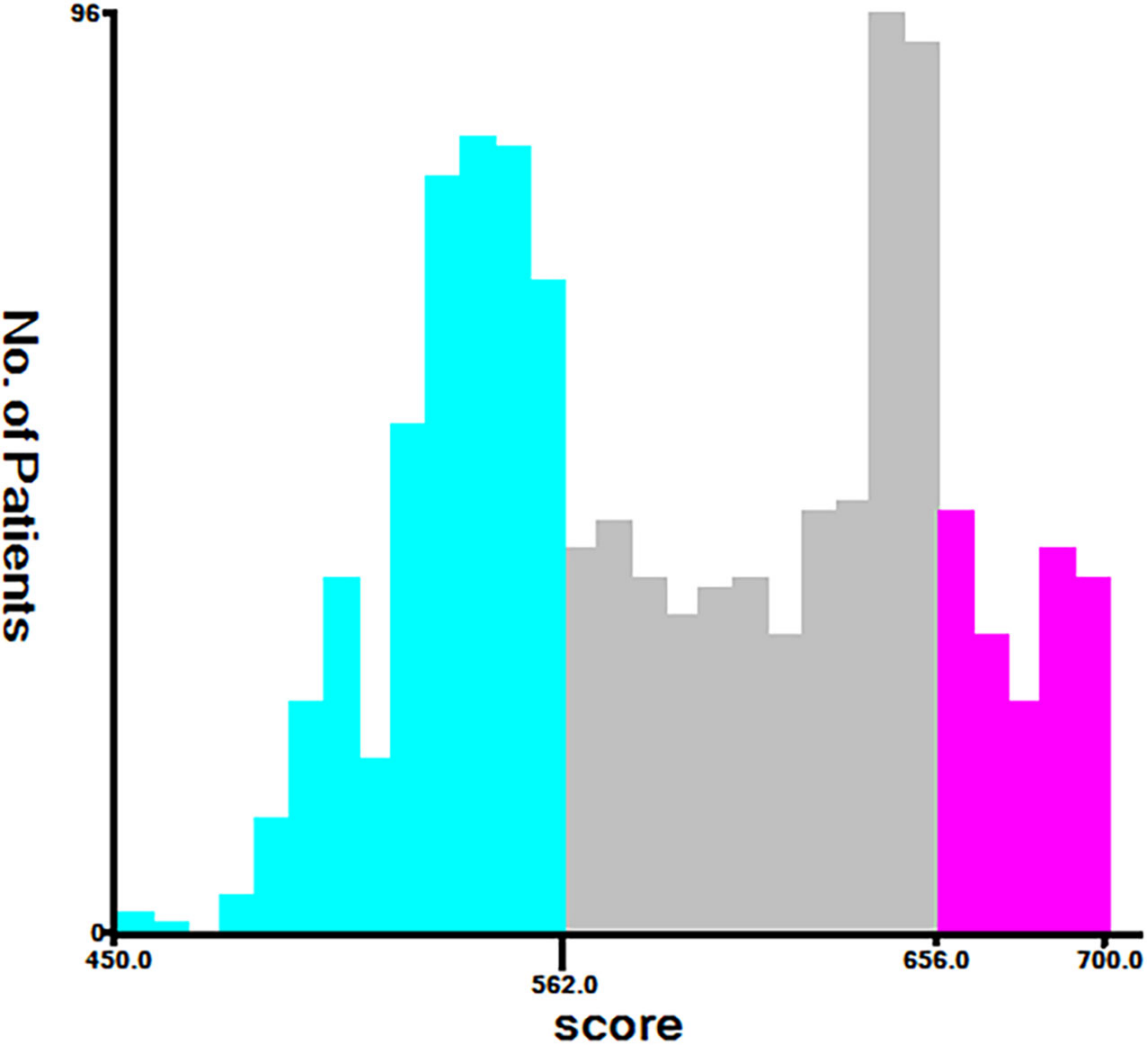
Cut-off point for risk stratification selected using X-tile.

**Figure 8:**
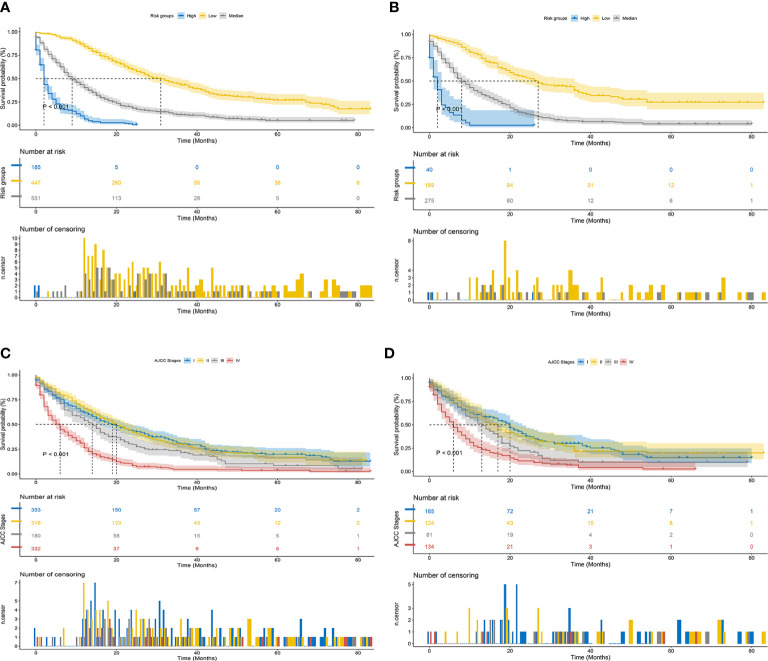
Kaplan–Meier CSS curves of patients with ECCA based on different criteria. **(A, B)** Kaplan–Meier CSS curves of the training and validation cohorts based on the new risk stratification system. **(C, D)** Kaplan–Meier CSS curves of the training and validation cohorts based on AJCC staging criteria.

Finally, risk stratification was performed based on the total points calculated by the nomogram. Patients with ECCA were divided into three risk groups, namely, low risk (total points <562), middle risk (562 ≤ total points < 656), and high risk (total points ≥ 656) (**Figure 7**). The Kaplan–Meier curve of CSS showed significant discrimination in these three risk groups, while the AJCC staging system had limited identification of low-risk and high-risk patients in the training and validation cohorts (**Figure 8**).

## Discussion

ECCA is a highly lethal epithelial malignancy with a poor prognosis, and the incidence of this cancer has increased in recent years (16). Several previous studies have focused on the prognostic factors of ECCA, including radical surgery (17), preoperative cholangitis, and lymph node metastasis (18). However, few studies have evaluated the prognosis of ECCA as a separate solid tumor until now. Therefore, a nomogram was constructed to predict the prognosis of patients with ECCA. The validation results of the nomogram showed excellent discrimination and calibration ability. Age, AJCC staging system, pathological grade, lymph nodes, treatment, chemotherapy, tumor size, tumor number, and marital status were independent prognostic factors (*P*<0.05) affecting patients with ECCA in this analysis, which was similar to the findings reported by Zhao et al. (19).

Older (especially >65 years old) had worse prognosis (HR=0.39; 95% CI=0.30-0.52; *P*<0.001). Kim et al. also showed significantly lower survival at age > 65 years (HR=1.32; 95% CI=1.09-1.60) (20). This phenomenon may be related to the poor tolerance of surgery or many underlying diseases in elderly individuals. In the present study, the tumor number, size, differentiation degree, and regional lymph nodes were independent prognostic factors, which was consistent with previous research. Zhang et al. suggested that patients with larger tumors and worse tumor differentiation were more likely to experience regional lymph node positivity and vascular invasion (21). In the present study, sex was an independent prognostic factor in the univariate analysis (*P*<0.001), and it was not statistically significant in the multivariate model (*P*=0.79). Previous studies have shown that sex is an independent prognostic factor and that male patients have shorter survival times than female patients (22, 23).

Surgery is the only cure for cholangiocarcinoma (24), and the present study indicated that patients were more likely to benefit from hepatectomy (HR=0.39; 95% CI=0.30-0.52; *P*<0.001) and liver transplantation (HR=0.34; 95% CI=0.25-0.46; *P*<0.001) than without any surgery. For chemotherapy, patients could also benefit from surgery (HR=2.09; 95% CI=1.79-2.44; *P*<0.001). According to the National Comprehensive Cancer Network (NCCN) guidelines, chemotherapy regimens mainly include fluoropyrimidine-based or gemcitabine-based chemotherapy (25). A phase III clinical trial has demonstrated that patients receiving postoperative adjuvant chemotherapy with capecitabine have improved overall survival (26). Based on these retrospective studies, we concluded that patients benefit from adjuvant chemotherapy (27–29) although radiotherapy had little impact on patient outcomes in the present analysis. In a SEER-based analysis, Vern-Gross et al. also found that adjuvant radiotherapy is not associated with improved long-term overall survival in patients with ECCA (30). However, there is some favorable evidence to support the application of radiotherapy in patients with ECCA (31–33).

More research has begun to focus on the prognostic impact of marital status on gastrointestinal tumors in recent years. For gastric, gallbladder, or cholangiocarcinoma, the risk of death in unmarried individuals (including divorced and widowed) is higher than that in married individuals (19, 34–36). In the present study, divorce was a poor prognostic factor (HR=1.24; 95% CI=1.05-1.47; *P*<0.001), which was consistent with previous findings. This result may be related to spouse companionship, spiritual support, and financial support.

Clinicians generally use the AJCC staging system to evaluate the prognosis of patients, but this staging system does not fully account for patients’ age, sex, marital status, and adjuvant treatment. However, a nomogram is a quantitative model integrating multiple factors, including demographic and clinical characteristics, with higher predictive accuracy and discriminatory ability to predict survival (15, 37–39). Comparison of the nomogram to the conventional AJCC staging system demonstrated that the nomogram had better predictive power and better clinical benefit. In the present study, we classified ECCA patients into low-, middle-, and high-risk groups according to the total points of the nomogram. The results of the Kaplan–Meier and Cox hazard ratio models indicated significant differences in CSS among these three groups. Because the high-risk group had a poor prognosis, more attention should be given to patients in this group.

This nomogram has some potential value in clinical practice. For example, it may better predict the prognosis of patients, promote the choice of postoperative treatment decisions (such as radiotherapy, chemotherapy, or immunotherapy), and help to develop and adjust the follow-up intervals to achieve individual monitoring of the disease. However, the present study had several limitations. For example, some data not published or missing in the database, such as CA19-9 levels, were excluded from the analysis. Tella et al. found that CA19-9 is a poor prognostic factor for OS in ECCA (HR: 1.72; 95% CI=1.462.02; *P*<0.01), and they considered that the inclusion of CA19-9 levels in the AJCC staging system helps physicians assess patient outcomes more accurately (NRI=46%; 95% CI=39-57%) (40). Second, these data were retrospective, leading to selection bias in the present study. In addition, conducting a multicenter large-scale prospective clinical study is challenging due to the rarity of the disease.

## Conclusion

Compared to the current AJCC staging system, our nomogram improves the ability to predict individual patient survival and shows consistent reliability and clinical utility in clinical evaluation. Further studies are needed to confirm our findings.

## Data availability statement

The datasets presented in this study can be found in online repositories. The names of the repository/repositories and accession number(s) can be found in the article/**Supplementary Material**.

## Author contributions

Conceptualization, FZ. Data curation, JH and XJ. Formal analysis, FZ and DY. Funding acquisition, XL. Investigation, FZ, DY and YD. Methodology, FZ. Project administration, XL. Resources, JH and XJ. Software, DY. Validation, DY. Visualization, XL. Writing–original draft, FZ. Writing–review & editing, YD and XL. FZ and DY contributed equally to this work and share first authorship. All authors contributed to the article and approved the submitted version

## Acknowledgments

Thanks to all the authors for their serious responsibility and dedication.

## Conflict of interest

The authors declare that the research was conducted in the absence of any commercial or financial relationships that could be construed as a potential conflict of interest.

## Publisher’s note

All claims expressed in this article are solely those of the authors and do not necessarily represent those of their affiliated organizations, or those of the publisher, the editors and the reviewers. Any product that may be evaluated in this article, or claim that may be made by its manufacturer, is not guaranteed or endorsed by the publisher.
