# Supplementary material for: Establishment and validation of a prognostic nomogram for extrahepatic cholangiocarcinoma
Source: Front Oncol. 2022 Nov 24;12:1007538. doi: 10.3389/fonc.2022.1007538 (PMC9730808; doi:10.3389/fonc.2022.1007538)
Supplement: Supplementary file 1 [file Table_1.docx]

Supplemental table 1: The differences between the AJCC staging system and nomogram.

|  | AJCC staging system | Nomogram |
| --- | --- | --- |
| C index | Low | Strong |
| ROC | Low | Strong |
| Prediction ability | Low | Strong |
| Ability to distinguish between different risk groups | Low | Strong |
| Others | Wide range of applications | Many variables were included, and the application was more complicated |
