# Supplementary material for: Establishment and validation of a prognostic nomogram for extrahepatic cholangiocarcinoma
Source: Front Oncol. 2022 Nov 24;12:1007538. doi: 10.3389/fonc.2022.1007538 (PMC9730808; doi:10.3389/fonc.2022.1007538)
Supplement: Supplementary file 2 [file Table_2.docx]

| **Variable** | Age | Race | Sex | Grade | Regional nodes | AJCC Stages | Size | Number | Treatment | Radiation sequence | Chemotherapy | Marital | Insurance |
| --- | --- | --- | --- | --- | --- | --- | --- | --- | --- | --- | --- | --- | --- |
| **Variance inflation factors** | 2.927 | 1.696 | 2.411 | 1.852 | 1.889 | 1.982 | 3.521 | 1.147 | 2.543 | 1.178 | 1.625 | 1.126 | 2.483 |

Supplemental table 2. The results of multicollinearity test.
